# Pediatric minor head trauma: do cranial CT scans change the therapeutic approach?

**DOI:** 10.6061/clinics/2016(10)09

**Published:** 2016-10

**Authors:** Felipe P Andrade, Roberto Montoro, Renan Oliveira, Gabriela Loures, Luana Flessak, Roberta Gross, Camille Donnabella, Andrea Puchnick, Lisa Suzuki, Rodrigo Regacini

**Affiliations:** I Universidade Anhembi Morumbi, Laboratório de Simulação, São Paulo/SP, Brazil; II Hospital Infantil Sabará, Departamento de Diagnóstico por Imagem, São Paulo/SP, Brazil; III Universidade Federal de São Paulo (UNIFESP), Departamento de Diagnóstico por Imagem, São Paulo/SP, Brazil

**Keywords:** Computed Tomography, Minor head trauma, Pediatric, CT Scan, Radiology

## Abstract

**OBJECTIVES:**

1) To verify clinical signs correlated with appropriate cranial computed tomography scan indications and changes in the therapeutic approach in pediatric minor head trauma scenarios. 2) To estimate the radiation exposure of computed tomography scans with low dose protocols in the context of trauma and the additional associated risk.

**METHODS:**

Investigators reviewed the medical records of all children with minor head trauma, which was defined as a Glasgow coma scale ≥13 at the time of admission to the emergency room, who underwent computed tomography scans during the years of 2013 and 2014. A change in the therapeutic approach was defined as a neurosurgical intervention performed within 30 days, hospitalization, >12 hours of observation, or neuro-specialist evaluation.

**RESULTS:**

Of the 1006 children evaluated, 101 showed some abnormality on head computed tomography scans, including 49 who were hospitalized, 16 who remained under observation and 36 who were dismissed. No patient underwent neurosurgery. No statistically significant relationship was observed between patient age, time between trauma and admission, or signs/symptoms related to trauma and abnormal imaging results. A statistically significant relationship between abnormal image results and a fall higher than 1.0 meter was observed (*p*=0.044). The mean effective dose was 2.0 mSv (0.1 to 6.8 mSv), corresponding to an estimated additional cancer risk of 0.05%.

**CONCLUSION:**

A computed tomography scan after minor head injury in pediatric patients did not show clinically relevant abnormalities that could lead to neurosurgical indications. Patients who fell more than 1.0 m were more likely to have changes in imaging tests, although these changes did not require neurosurgical intervention; therefore, the use of computed tomography scans may be questioned in this group. The results support the trend of more careful indications for cranial computed tomography scans for children with minor head trauma.

## INTRODUCTION

Head Trauma is responsible for more than 1.0 million emergency room (ER) visits, 95,000 hospitalizations, 7,000 deaths and 29,000 permanent disabilities, and in the United States (US) alone, costs surpass US $1.0 billion in hospital care annually 1-3. It is the leading trauma related morbidity/mortality in children worldwide and accounts for most indications of computed tomography (CT) scans in this group 4.

The "golden hour" concept in the trauma scenario is common knowledge in the medical community 2. Clinical signs of intracranial brain injury (IBI) in children are less reliable, which increases the use of CT scans, which are a highly sensitive imaging tests that can detect early IBI or help to safely discharge patients with head traumas 1-4.

However, up to 97% of CT findings are negative and less than 1% require neurosurgical intervention in this scenario 1,5,6. The short-term disadvantages of indiscriminate CT use include higher health costs, more sedation procedures, increased length of stay in the ER and additional dissatisfaction of parents. The major long-term disadvantage of CT use is premature exposure to ionizing radiation, which is associated with an increased risk of cancer and mortality 4. Despite these disadvantages, the use of CT scans after minor head trauma in children more than doubled from 1995 to 2005 7,8.

To establish clinical criteria for neuroimaging after minor head trauma in pediatric patients, Kuppermann et al. proposed “The Pediatric Emergency Care Research Network (PECARN)”, the largest cohort study on the topic, which has been used by other authors a reference standard that objectively defines clinically important brain injuries. However, significant variations in neuroimaging indications after mild pediatric head trauma still persist, with rates ranging from 5 to 70% in the US 3,5. Recent studies support the idea of observation in the ER in favor or avoiding CT scans, and the PECARN requires more clinical evidence to better define important brain injuries 9,10.

Although CT scans represent only approximately 11% of all radiological images in the US and 4% in Europe, dose levels administered in CT scans may have an influence on the stimulation of genetic mutations and carcinogenesis 11, and this information should be known and available to patients and their physicians 12.

The effective dose of ionizing radiation is primarily used to compare the cumulative risk (stochastic effect) associated with exposure to ionizing radiation and this risk requires special attention, particularly when repeated examinations are performed 13.

This study aims to include not only the clinical variables that could affect decision-making about cranial CT scans in pediatric minor head traumas but also to address one of the most important issues that involves performing an imaging test: does the result change the therapeutic approach and justify the risks associated with exposure to ionizing radiation?

## MATERIALS AND METHODS

A cross-sectional study was performed at Hospital Infantil Sabará, a tertiary care pediatric hospital in São Paulo, Brazil. The researchers reviewed the medical records of patients admitted to the ER with head trauma who underwent CT scans in the years 2013 and 2014 and included only those with a score on the Glasgow Coma Scale (GCS) ≥13, corresponding to a minor head trauma. We did not exclude patients with trauma in other areas of the body associated with head trauma or those who could be identified as potential victims of abuse.

Information on demographics, physical examination findings, symptoms, mechanism and time of trauma, medical management and discharge data were obtained from hospital records. The CT scan interpretations were obtained from the final reports of attending radiologists. Subgaleal hematoma or extracranial changes were not considered as relevant findings. The main relevant changes considered were extra-axial hemorrhage (epidural, subdural and arachnoid hemorrhage), intra-axial hemorrhage (intracerebral or intraventricular hemorrhage) and fractures. Any other possible intracranial changes were also considered including pneumocephalus, brain edema and cerebral herniation, among others.

The variables collected for analysis of their relationship with changes in the therapeutic approach after imaging were age, gender, length of loss of consciousness, mechanism of trauma, vomiting, seizure, nausea, headache, drowsiness, dizziness, cranial hematomas, cranial laceration and visual alterations.

Changes in the therapeutic approach after CT scans included the following: neurosurgical intervention within 30 days, hospitalization, >12 hours of observation, and neuro-specialist evaluation.

The dose-length product (DLP) was used to determine the effective dose according to the new International Commission on Radiological Protection (ICRP) recommendations 12,14. All CT scans was performed using a low-dose protocol (80 kV).

Statistical analyses were performed with the Statistical Package for Social Sciences (SPSS), version 16.0, IBM®, USA.

All variables were descriptively analyzed. All data were summarized as numbers and percentages (%) or means and standard deviations according to the variable type.

The chi-square test or Fisher's exact test were used to determine the association between the physical examination findings, symptoms, mechanism of trauma, medical management and discharge data and the image findings. Student's t test for independent variables was used to determine the association between the timing of trauma and hospitalization and the image findings. The results were considered significant when the *p*-value was less than 0.05 (*p*<0.05).

### Ethics

This study was conducted according to the Declaration of Helsinki and was approved by the Hospital Infantil Sabará Institutional Review Board.

## RESULTS

A total of 1,006 children comprised our study population, which included patients aged 0 to 17.83 years, with a mean age of 3.9±3.7 years, including 545 (54.2%) boys and 461 (45.8%) girls. The trauma-admission time ranged from 0.1 to 336.0 hours (mean of 15.0±35.0 hours). The mean length of stay in the hospital was 9.0±21.4 hours, ranging from 0.30 to 192.0 hours. Table 1 shows the frequency of signs and symptoms and the probability (%) of any abnormal imaging results related to these variables. Regarding the trauma mechanism, 81.1% of the injuries were falls, of which 40.2% corresponded to falls of ≤1 m.

The following signs and symptoms were surveyed: vomiting, nausea, headache, drowsiness, dizziness, visual alterations, loss of consciousness, convulsion, laceration, and penetration of objects. No type of injury described in the tables as other was significantly associated with abnormal imaging results. In the imaging studies, 887 (88.2%) patients underwent only a CT scan. The other 119 patients completed imaging examinations consisting of x-ray examinations (9.4%), ultrasound examinations (0.6%), Magnetic Resonance Imaging (MRI) (0.9%), or combined x-ray and ultrasound examinations (0.9%).

Of the 1006 patients undergoing imaging examinations, only 101 (10.0%) showed abnormal imaging findings. Cranial fractures were the most common finding (Table 2). Most patients were discharged after imaging examinations (84.7%) and no patient underwent neurosurgery (Table 3).

No significant differences between patients with and without abnormal CT results were found with regard to patient age (*p*=0.876) or trauma-admission time (*p*=0.084). Changes in the therapeutic approach after performing CT scans occurred in 15.6% of all patients in this study, with 64.3% consisting of patients with abnormal imaging results, and 10.6% were performed in those with normal results. A statistically significant change in the therapeutic approach was demonstrated in cases with a neuro-specialist referral (*p*<0.0001) and the need for >12 hours of observation (*p*=0.001).

Subjects were divided in two groups according to trauma-admission time (≤2 hours and >2 hours) and then analyzed according to imaging findings. Of the 101 patients with abnormal imaging findings, 33.7% presented a trauma-admission time ≤2 hours. A significant difference was not observed between patients with and without imaging abnormalities with regard to trauma-admission time (*p*=0.979).

A statistically significant association was found between abnormal imaging results and fall height (Tables 2 and 4). Among the 101 patients with abnormal imaging results, 35.6% suffered falls of ≤1 m in height, and 14.9% suffered falls of >1 m in height. Of all children who suffered falls of ≤1 m, 8.9% presented abnormal imaging results; however, of all children who suffered falls of >1 m, 16.3% were revealed to have abnormal imaging findings.

Among the 101 children with abnormal imaging results, 48.5% were hospitalized, 15.8% underwent observation and 35.6% were dismissed (Table 3). In total, 112 patients were referred to a specialist; of these, 41.1% showed abnormalities on imaging and 58.9% had a normal CT scan.

Information about the DLP was recovered for 357 (35.5%) CT scans. The DLP ranged from 13.2 to 797.8 mGy·cm (mean of 401.0±163.1 mGy·cm), which is equivalent to an effective dose of 2.0 mSv (0.1-6.8 mSv) and corresponds to an estimated additional cancer risk of 0.05%.

## DISCUSSION

This two-year cross-sectional study showed that the time between trauma and admission and clinical data, such as vomiting, nausea, headache, drowsiness, dizziness, visual disturbances, loss of consciousness, seizures, laceration and penetration of objects had no significant effect in predicting possible changes in cranial CT findings after minor head trauma in pediatric patients. No significant association was found between abnormal imaging results and patient age; therefore, unlike other studies 5,9,12,15,16, we did not categorize patients as infants and children. Furthermore, the only variable that showed a statistically significant association with abnormal imaging results and a change in the medical approach was a fall from a height of >1.0 m.

According to our findings, standard clinical references used in previous studies (PECARN) 3, such as altered mental status, loss of consciousness, severe headache and vomiting, did not contribute to the decision to perform a CT scan in the context of head trauma. Some difficulties in the interpretation of those variables may be present. For instance, variations in mental status could be due to several reasons, such as an undiagnosed pathology, the mechanism of injury was frequently not witnessed, or the report of patient loss of consciousness was not clear. In addition, multiple studies 17 that exclusively examined vomiting and headache as criteria for performing a CT scan missed an important IBI. Approximately one quarter of preverbal children hospitalized for head trauma are victims of child abuse; therefore, information about the injury is even less reliable 10,15.

In addition, a review of the literature reveals no consensual definition of what constitutes a “minor head trauma”. Usually, different studies refer to minor trauma as cases in which children have GCS scores ranging from 13 to 15, although this slight variation increases the risk of IBI from approximately 2-3% in children with a GCS score of 15 to 7-8% in patients with a GCS score of 14 and to nearly 25% in children with a GCS score of 13 3,16.

Skull fracture was the most prevalent imaging abnormality found in our study. The results corroborate the well-established fact that no correlation exists between skull fracture and IBI 18 because, in most cases, fractures due to mild trauma are small and do not produce significant misalignment. Thus, we discourage the use of cranial x-rays in pediatric minor head trauma patients, as it will not change the subsequent treatment.

Most children who were hospitalized, under observation >12 hours, or referred to a neuro-specialist showed some abnormality on imaging tests, and the differences were significant for the last two approaches. When CT scans present any changes, even without clinical significance, the attending physician tends to place the child under observation or request a neuro-specialist evaluation. Thus, concerns about exposure to ionizing radiation must be weighed against the benefits of these interventions because surgery is not typically required.

Some patients who were referred to the neuro-specialist or were hospitalized returned later to undergo a control CT scan. This follow-up image was not included in our data and can be considered a limitation of this study.

The increase in the baseline risk of long-term malignancy is the main argument in favor of more careful use of cranial CT scans in the context of minor head trauma, particularly in the pediatric population, in which the tissues are more sensitive to ionizing radiation 19,20,21. These patients are subjected to repeated CT scans and have a longer lifetime to manifest a radiation-induced malignancy 2,6. However, low ionizing radiation protocols expose children to very low doses of radiation, and although not part of the scope of this paper, a CT scan in the context of trauma can help reassure parents of pediatric trauma victims, reduce observation time or the probability of hospitalization, and allow the attending physician to safely discharge the child.

CT scans performed after pediatric minor head injury did not show clinically significant abnormalities that could lead to neurosurgical indications.

The mechanism of trauma plays a more important role than isolated clinical signs and symptoms, patient age, or trauma-admission time in changing the medical approach in pediatric minor head trauma cases. According to the findings of this article, victims who fell more than 1.0 m were more likely to have changes in imaging tests, although this did not necessarily require neurosurgical intervention; therefore, the use of CT scans may be questioned in this group.

The results of this study support the trend of more cautious indications for cranial CT scans for children with minor head trauma. Imaging protocols based on clinical data in the context of pediatric minor head trauma require greater external validation to ensure reliability and applicability because the variables involved may be subjective.

## AUTHOR CONTRIBUTIONS

Andrade FP designed the project, performed data collection, and wrote the manuscript. Neto RM designed the project and performed the data collection. Oliveira R performed the data collection and statistical analysis. Loures G revised the manuscript and performed data collection. Flessak L, Donnabella C performed data collection and revised the manuscript. Gross R performed data collection and statistical analysis. Puchnick A assisted in the writing of the manuscript and performed statistical analysis. Suzuki L designed the project, performed data collection and analyzed the CT images. Regacini R designed the project, assisted in the discussion and conclusion and analyzed the CT images.

## Figures and Tables

**Table 1:** Frequency of clinical history and physical exam findings in victims of minor head trauma and their correlation with abnormal imaging findings.

Finding	Frequency	%	CT scan abnormality		
			Yes (%)	No (%)	*p*-Value
Vomiting	337	33.5	29 (8.6%)	308 (91.4%)	0.318
Nausea	64	6.4	4 (6.3%)	60 (93.8%)	0.391
Headache	203	20.2	17 (8.4%)	186 (91.6%)	0.434
Drowsiness	352	35.0	33 (9.4%)	319 (90.6%)	0.661
Dizziness	67	6.7	3 (4.5%)	64 (95.5%)	0.141
Visual alterations	30	3.0	0 (0%)	30 (100%)	0.121
Loss of consciousness	72	7.2	7 (9.7%)	65 (90.3%)	0.926
Convulsion	31	3.1	2 (6.5%)	29 (93.5%)	0.761
Laceration	55	5.5	7 (12.7%)	48 (87.3%)	0.487
Subgaleal hematoma	397	39.5	62 (15.6%)	397 (84.4%)	<0.0001
Object penetration	4	0.4	1 (25.0%)	3 (75.0%)	0.345
Other injuries	177	17.6	21 (11.9%)	156 (88.1%)	0.408

**Table 2:** Correlation between imaging findings and mechanism of trauma.

Mechanism of trauma	Imaging findings				
	Extra-axial hemorrhage	Intra-axial hemorrhage	Intracranial hemorrhage and fracture	Fracture	Other
Fall from own height	7	2	10	3	2
Fall of ≤1 m	6	2	4	24	0
Fall of >1 m	0	1	5	8	1
Fall from stairs	1	0	0	2	0
Other	9	4	6	4	
Total	23	9	25	41	3

Pearson Chi-Square *p*=0.050

**Table 3:** Frequency of therapeutic approach adopted for victims of minor head trauma.

Approach	Frequency	%	CT scan abnormality	
			Yes (%)	No (%)
Dismissed	849	84.7	36 (4.2%)	813 (95.8%)
Observation	59	5.9	16 (27.1%)	43 (72.9%)
Admitted	98	9.7	49 (50.0%)	49 (50.0%)
Surgery	0	0	—	—
Return to ER	46	4.6	9 (19.6%)	37 (80.4%)
Specialist referral	112	11.1	46 (41.1%)	66 (58.9%)

**Table 4:** Frequency of therapeutic approach adopted for victims of minor head trauma and correlation with abnormal imaging findings.

Approach	Frequency	%	CT scan abnormality		
			Yes (%)	No (%)	*p*-Value
Fall from own height	302	30.0	24 (7.9%)	278 (92.1%)	0.170
Fall of ≤1 m	404	40.2	36 (8.9%)	368 (91.1%)	0.338
Fall of >1 m	92	9.1	15 (16.3%)	77 (83.7%)	0.044
Fall from stairs	18	1.8	3 (16.7%)	15 (83.3%)	0.414
Other	190	18.9	23 (12.1%)	190 (87.9%)	0.286
